# Synthesis and Antiplasmodial Evaluation of Analogues Based on the Tricyclic Core of Thiaplakortones A–D

**DOI:** 10.3390/md13095784

**Published:** 2015-09-15

**Authors:** Brett D. Schwartz, Mark J. Coster, Tina S. Skinner-Adams, Katherine T. Andrews, Jonathan M. White, Rohan A. Davis

**Affiliations:** 1 Eskitis Institute for Drug Discovery, Griffith University, Nathan, Qld 4111, Australia; E-Mails: b.schwartz@griffith.edu.au (B.D.S.); m.coster@griffith.edu.au (M.J.C.); t.skinner-adams@griffith.edu.au (T.S.S.); k.andrews@griffith.edu.au (K.T.A.); 2 School of Chemistry and Bio21 Institute, University of Melbourne, Parkville, Vic 3052, Australia; E-Mail: whitejm@unimelb.edu.au

**Keywords:** synthesis, thiaplakortone, regioisomer, tricyclic, natural product scaffold, X-ray, crystal, *Plasmodium falciparum*, antiplasmodial, cytotoxicity

## Abstract

Six regioisomers associated with the tricyclic core of thiaplakortones A–D have been synthesized. Reaction of 1*H*-indole-4,7-dione and 1-tosyl-1*H*-indole-4,7-dione with 2-aminoethanesulfinic acid afforded a regioisomeric series, which was subsequently deprotected and oxidized to yield the tricyclic core scaffolds present in the thiaplakortones. All compounds were fully characterized using NMR and MS data. A single crystal X-ray structure was obtained on one of the *N*-tosyl derivatives. All compounds were screened for *in vitro* antiplasmodial activity against chloroquine-sensitive (3D7) and multidrug-resistant (Dd2) *Plasmodium falciparum* parasite lines. Several analogues displayed potent inhibition of *P. falciparum* growth (IC_50_ < 500 nM) but only moderate selectivity for *P. falciparum* versus human neonatal foreskin fibroblast cells.

## 1. Introduction

**Figure 1:**
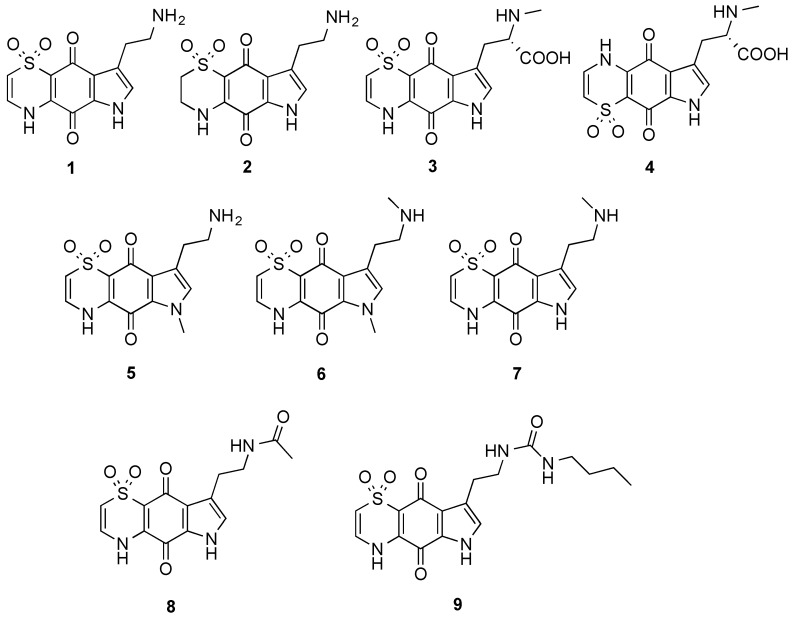
Chemical structures of the natural products thiaplakortones A–D (**1**–**4**) and some of the previously synthesized thiaplakortone A analogues (**5**–**9**).

The marine natural products, thiaplakortones A–D (**1**–**4**), were first reported in 2013 as part of a Medicines for Malaria Venture sponsored research project that aimed to discover new antiplasmodial agents from nature (Figure 1) [1]. These unique thiazine-derived secondary metabolites were obtained from the organic extract from the Great Barrier Reef sponge *Plakortis lita*, and all were shown to inhibit the *in vitro* growth of *Plasmodium falciparum*. Thiaplakortone A (**1**) was the most active with *in vitro* IC_50_ values of 6.6 and 51 nM against multidrug-resistant (Dd2) and chloroquine-sensitive (3D7) *P. falciparum* lines, respectively [1]*.* Due to supply issues initially curtailing *in vivo* malaria studies, total syntheses of thiaplakortones A and B were undertaken and the first total synthesis of **1** and **2**, along with a series of mono- and di-methyl analogues (**5**–**7**) was subsequently reported and some preliminary structure-activity relationships (SAR) ascertained (Figure 1) [2]. While *in vivo* toxicity effects for several of the synthetic compounds indicated potential liabilities associated with this structure class, the limited number of analogues investigated made it difficult to assess their true potential as antiplasmodial leads [2]. In order to more thoroughly explore this compound class a larger analogue library based on the thiaplakortone A scaffold was recently undertaken and reported [3]. This 38-membered library consisted of a series of amide and urea analogues based on the thiaplakortone A natural product scaffold. Several analogues showed potent *in vitro P. falciparum* growth inhibition (IC_50_ < 500 nM) and good selectivity for *P. falciparum* versus human neonatal foreskin fibroblast (NFF) cells (selectivity index >100) [3]. Furthermore, analogues **8** and **9** displayed good metabolic stability and solubility, and when administered subcutaneously to mice plasma concentrations remained >0.2 µM for 8 h. Analogues **8** and **9** were also well tolerated in mice after subcutaneous administration of 32 mg/kg twice daily for 4 d. In addition, using this dosing protocol blood stage *P. berghei* parasitemia was suppressed by 52% for **8** and 26% for **9**, relative to controls [3]. In order to further investigate the thiaplakortone core, we have recently undertaken synthetic studies that resulted in the removal of the ethylamine side-chain present in thiaplakortones A and B in order to determine the biological implications of the –CH_2_CH_2_NH_2_ moiety. Herein we report the total synthesis of several side-chain truncated regioisomers associated with the tricyclic core of thiaplakortones A–D, along with their *in vitro* antiplasmodial activity and mammalian cell toxicity.

## 2. Results and Discussion

### 2.1. Chemistry

**Scheme 1:**
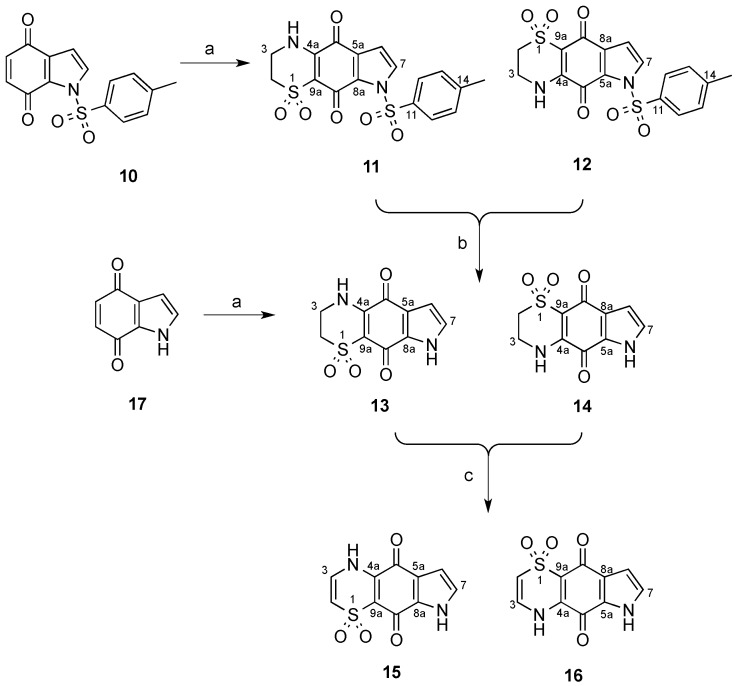
Synthesis of compounds **11**–**16** in the thiaplakortone tricyclic series. (**a**) 2-aminoethanesulfinic acid, H_2_O, MeCN; (**b**) NaHCO_3(aq)_, MeOH, reflux 2.5 h; (**c**) KOH_(aq)_, MeOH, O_2_.

The synthesis of the tricyclic core thiaplakortone isomers **11**–**16** commenced with the generation of 1-tosyl-1*H*-indole-4,7-dione (**10**), which was accessible via known procedures (Scheme 1) [4,5,6]. Condensation of **10** with 2-aminoethanesulfinic acid [2,7] furnished the regiomeric tricyclic systems **11** and **12** in an 11 to 1 ratio (Scheme 1). Separation of this mixture was not possible by silica flash chromatography however reversed-phase C_18_ HPLC (MeOH-H_2_O-0.1%TFA) enabled separation of the two regioisomers. Confirmation of the chemical structures of **11** and **12** was supported following extensive 1D and 2D NMR data analysis.

**Figure 2:**
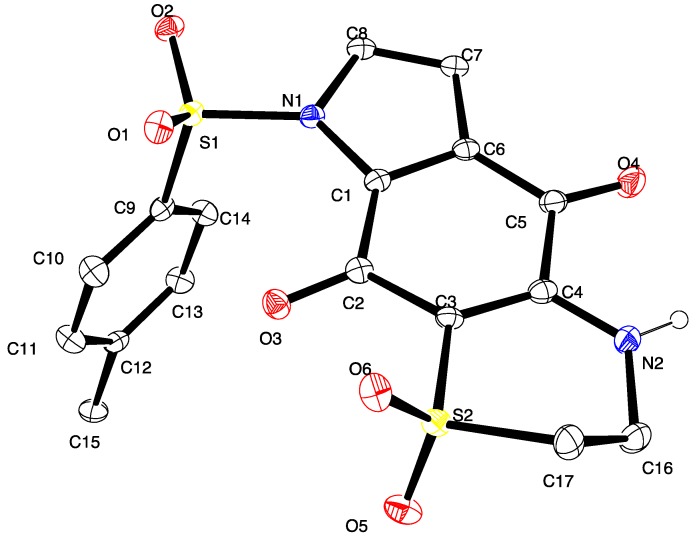
ORTEP diagram showing one independent molecule for compound **11**; ellipsoids are at the 30% probability level.

Furthermore, a crystal suitable for X-ray analysis was obtained on the major regioisomer **11** (Figure 2) confirming the NMR-assigned structure and establishing the regiochemistry of subsequent compounds in the tricyclic series. Of note, compound **11** crystallized with two molecules in the asymmetric unit; the second molecule displayed disorder (*ca*. 13%) in the thiazine dioxide ring (see supplementary data).

Subjection of the mixture of tosyl derivatives **11** and **12** to mild alkaline hydrolysis afforded a sufficient quantity of only compound **13** after purification by reversed-phase HPLC (MeOH-H_2_O-0.1%TFA).

In an attempt to reverse the regioselectivity observed during the condensation of 2-aminoethanesulfinic acid with **10** and acquire suitable amounts of isomer **14**, the parent, non-tosyl protected system, 1*H*-indole-4,7-dione **17** was prepared according to literature procedures [5,8,9] and exposed to 2-aminoethanesulfinic acid. Gratifyingly, in this system, the opposite regioselectivity was observed and the respective tricyclic systems **13** and **14** were formed in a 1 to 3.3 ratio following analysis of the ^1^H-NMR spectrum. Purification of this mixture by C_18_ HPLC (MeOH-H_2_O-0.1%TFA) enabled **14** to be isolated in sufficient amounts for biological testing. Oxidation of the mixture of **13** and **14** by protocols previously reported [2] afforded a mixture that was subjected to C_18_ HPLC (MeOH-H_2_O-0.1%TFA) and yielded the pure compounds **15** and **16**.

### 2.2. Biology and Structure-Activity Relationships

All compounds (**10**–**17**) were tested for *in vitro* antiplasmodial activity against chloroquine-sensitive (3D7) and multidrug-resistant (Dd2) *P. falciparum* parasite lines, and for mammalian toxicity using human neonatal foreskin fibroblast (NFF) cells. The simple indolequinones, **10** and **17**, were essentially inactive (Table 1), highlighting the importance of the 1,1-dioxo-thiazine subunit for antiplasmodial activity. The original thiaplakortone report [1] showed that unsaturation of the thiazine ring conferred enhanced antiplasmodial activity. This trend was also observed in the side-chain truncated compounds, with the unsaturated compounds, **15** and **16** displaying greater potency than their saturated counterparts, **13** and **14**, respectively. Specifically, exchanging the saturated system of **13** and **14** with the unsaturated motif present in **15** and **16** increased 3D7 activity by 23.9- and 27.3-fold, respectively; a similar SAR trend was also observed for this series towards the Dd2 line.

**Table 1:** Biological Data for Compounds **10**–**17**.

	Mean IC_50_ ± SD (nM)			
Compound	3D7 *^a^*	Dd2 *^b^*	NFF *^c^*	SI *^d^*
**10**	18,200 ± 2600	11,100 ± 4100	7600 ± 1200	0.4–0.7
**11**	546 ± 119	509 ± 309	1400 ± 700	2.6–2.8
**12**	834 ± 89	607 ± 158	19,000 ± 11,000	22.8–31.3
**13**	7500 ± 900	3800 ± 400	39,000 ± 4200	5.2–10.3
**14**	6900 ± 700	3700 ± 500	69,600 ± 5900	10.1–18.8
**15**	313 ± 84	129 ± 3.9	2800 ± 400	8.9–21.7
**16**	252 ± 35	127 ± 8.6	4600 ± 800	18.2–36.2
**17**	13,500 ± 6700	11,500 ± 6500	4700 ± 100	0.3–0.4
**CQ** *^e^*	7.8 ± 2.7	45 ± 10	36,500 ± 6000	811.1–4679.5

*^a^* 3D7 = *P. falciparum* chloroquine-sensitive line; *^b^* Dd2 = *P. falciparum* multidrug-resistant line; *^c^* NFF = neonatal foreskin fibroblast cells; *^d^* SI = selectivity index = NFF cell-line IC_50_/*P. falciparum* IC_50_; *^e^* CQ = chloroquine (positive control).

The regiochemistry of the thiazine moiety in the original thiaplakortone report was shown to have minimal influence on the overall antiplasmodial activity and selectivity [1]. Reversal of the thiazine orientation in thiaplakortones C (**3**) and D (**4**) only showed an antiplasmodial activity increase of 1.1-fold towards both the 3D7 and Dd2 lines. In a similar manner to the earlier report, the current studies showed minimal differences in parasite potency between the side-chain truncated regioisomeric pairs, **11** and **12**, **13** and **14**, and **15** and **16**. However, when comparing NFF toxicity of the regioisomers (**11** *vs.* **12**; **13** *vs.* **14**; **15** *vs.* **16**) a clear trend was observed, with the thiazine regiochemistry present in **12**, **14** and **16** conveying reduced cytotoxicity ranging from 1.6- to 13.6-fold. Furthermore, the reduction in toxicity improved the selectivity indices for analogues **12**, **14** and **16**.

Biological data for compounds **11** and **12** identified that *N*-tosylation of the pyrrole moiety is well tolerated and improves antiparasitic activity, even in the absence of thiazine unsaturation. It is interesting to note that, consistent with the thiaplakortone natural products [1], the most active side-chain truncated analogues, **15** and **16**, are more potent against the drug-resistant line (Dd2) than the chloroquine-sensitive line (3D7). While it is clear that the ethylamine side-chain present in thiaplakortones A and B translates to more potent and selective antiplasmodial agents, the current study shows that the tricyclic core motif present in **11**–**16** represents a minimum antiplasmodial pharmacophore for the thiaplakortone chemotype.

In order to assess the drug-like properties of compounds **10**–**17**, *in silico* physicochemical parameters (Table 2) were calculated using ChemAxon MarvinSketch software (with calculator plugins) (http://www.chemaxon.com) and the data compared to Lipinski’s drug-like “Rule of Five” [10]. All compounds complied with Lipinski’s rules (LogP < 5, HBA < 10, HBD < 5, MW < 500). In addition, all compounds had relatively low LogD_7.4_ values (except compound **10**), and had appropriate polar surface area (PSA) values for membrane penetration.

**Table 2:** *In silico* physicochemical parameters for compounds **10**−**17** *^a^*.

Compound	MW	LogP	HBA	HBD	PSA (Å^2^)	LogD_7.4_
**10**	301	1.5	4	0	82	1.9
**11**	406	−1.6	7	1	136	−0.2
**12**	406	−1.6	7	1	136	−0.2
**13**	252	−3.3	5	2	104	−1.6
**14**	252	−3.3	5	2	104	−1.6
**15**	250	−2.9	5	2	104	−1.5
**16**	250	−2.9	5	2	104	−1.5
**17**	147	−0.2	2	1	50	0.5

*^a^ In silico* calculations performed using ChemAxon MarvinSketch software (with calculator plugins). MW = molecular weight (Da); HBA = H-bond acceptors; HBD = H-bond donors; PSA = polar surface area.

## 3. Experimental Section

### 3.1. General

Melting points were recorded on a capillary melting point apparatus and are uncorrected. Unless otherwise specified, ^1^H and ^13^C-NMR spectra were recorded at 30 °C in DMSO-*d*_6_ on a Varian INOVA 500 or 600 NMR spectrometer. The ^1^H- and ^13^C-NMR chemical shifts were referenced to the solvent peak for DMSO-*d*_6_ at δ_H_ 2.50 and δ_C_ 39.5. LRESIMS was obtained from LC-MS data generated using a Waters Alliance 2790 HPLC equipped with a Waters 996 photodiode array detector and an Alltech evaporative light scattering detector that was attached to a Water ZQ mass spectrometer. HRESIMS were recorded on a Bruker (Billerica, MA, USA) MicrOTof-Q spectrometer (Dionex UltiMate 3000 micro LC system, ESI mode). Analytical thin layer chromatography (TLC) was performed on aluminum-backed 0.2 mm thick silica gel 60 F_254_ plates as supplied by Merck (Frankfurt, Germany). Eluted plates were visualized using a 254 nm UV lamp and/or by treatment with a suitable dip followed by heating. These dips included phosphomolybdic acid:Ce(SO_4_)_2_:H_2_SO_4_ (conc.):H_2_O (37.5 g:7.5 g:37.5 g:720 mL) or KMnO_4_:K_2_CO_3_:5% NaOH aqueous solution:H_2_O (3 g:20 g:5 mL:300 mL). Flash chromatographic separations were carried out following protocols defined by Still *et al*., [11] with silica gel 60 (40–63 mm, supplied by GRACE, Baulkham Hills, NSW, Australia) or amino bonded silica gel (Davisil^®^) as the stationary phase and using the AR- or HPLC-grade solvents indicated. Semi-preparative HPLC work was performed using a Waters 600 pump and 966 PDA detector, a Gilson 715 liquid handler and a C_18_-bonded silica Betasil 5 μm 143 Å column (21.2 mm × 150 mm). Alltech sample preparative C_18_-bonded silica (35–75 μm, 150 Å) and an Alltech stainless steel guard cartridge (10 mm × 30 mm) were used for pre-adsorption and HPLC work. A Phenomenex C_18_-bonded silica Luna 3 μm 100 Å (4.6 mm × 50 mm) column was used for LC-MS studies. All compounds were analyzed for purity using LC-MS and shown to be >95% pure, unless otherwise stated. Starting materials and reagents were available from the Sigma-Aldrich (St. Louis, MO, USA), Merck (Frankfurt, Germany), AK Scientific Inc. (Union City, CA, USA), Matrix Scientific Chemical (Columbia, SC, USA) and were used as supplied. MeOH and CH_2_Cl_2_ were dried using a glass contour solvent purification system that is based upon a technology originally described by Grubbs *et al*. [12]. Where necessary, reactions were performed under a nitrogen atmosphere and glassware was heated in an oven at 140 °C then dried under vacuum prior to use. Compounds for biological studies were placed under high vacuum (0.05 mmHg) for several hours before testing to remove trace, residual solvents.

### 3.2. Synthesis of N-Tosyl Regioisomers **11** and **12**

A solution of *N*-tosyl-1*H*-indole-4,7-dione (500 mg, 1.66 mmol) in MeCN (80 mL) was treated with a solution of 2-aminoethanesulfinic acid (236 mg, 2.16 mmol) in H_2_O (50 mL) in one portion. The mixture was stirred for 20 h open to the atmosphere and then H_2_O was removed by rotary evaporation and the resulting solid collected by vacuum filtration. The crystals were washed with H_2_O (20 mL) then dried to afford a crude ~1:11 mixture of regioisomers **12** and **11**, respectively. This material (236 mg) was pre-adsorbed to C_18_-bonded silica (1 g) overnight, then packed into a guard cartridge that was subsequently attached to a C_18_-bonded silica semi-preparative HPLC column. Isocratic HPLC conditions of 90% H_2_O (0.1% TFA)/10% MeOH (0.1% TFA) were employed for the first 10 min, then a linear gradient to MeOH (0.1% TFA) was run over 40 min, followed by isocratic conditions of MeOH (0.1% TFA) for a further 10 min, all at a flow rate of 9 mL/min. Sixty fractions (60 × 1 min) were collected by time from the start of the HPLC run. All UV active fractions were analyzed by ^1^H-NMR spectroscopy and MS, and identical fractions were combined. This afforded **12** (7.6 mg, 1%, ^t^R = 37.0–38.0 min) and **11** (69 mg, 10%, ^t^R = 53.0–60.0 min). X-ray quality crystals of **11** were obtained through slow evaporation using a H_2_O/MeOH (1:9) mix.

Compound **11**: Dull orange crystals (H_2_O/MeOH); mp > 300 °C; ^1^H-NMR (600 MHz, DMSO-*d*_6_) δ_H_ 2.42 (3H, s, H-15), 3.27–3.29 (2H, m, H-2), 3.76–3.78 (2H, m, H-3), 6.83 (1H, d, *J* = 3.4 Hz, H-6), 7.50 (2H, d, *J* = 8.2 Hz, H-13), 7.90 (2H, d, *J* = 3.4 Hz, H-7), 8.01 (2H, d, *J* = 8.2 Hz, H-12), 9.01 (1H, br s, H-4); ^13^C-NMR (150 MHz, DMSO-*d*_6_) δ_C_ 21.2 (C-15), 39.3 (C-3), 48.2 (C-2), 107.5 (C-6), 108.2 (C-9a), 126.7 (C-5a), 128.6 (2C, C-12), 129.3 (C-7), 129.8 (2C, C-13), 130.8 (C-8a), 133.5 (C-11), 146.2 (C-14), 146.6 (C-4a), 167.1 (C-9), 175.1 (C-5); (+)-LRESIMS *m*/*z* (rel. int.) 407 (100) [M + H]^+^; (−)-LRESIMS *m*/*z* (rel. int.) 405 (100) [M − H]^−^; (+)-HRESIMS *m*/*z* 429.0200 [M + Na]^+^ (calcd for C_17_H_14_N_2_NaO_6_S_2_, 429.0185).

Compound **12**: Bright orange amorphous solid; ^1^H-NMR (600 MHz, DMSO-*d*_6_) δ_H_ 2.41 (3H, s, H-15), 3.26–3.28 (2H, m, H-2), 3.75–3.77 (2H, m, H-3), 6.81 (1H, d, *J* = 3.2 Hz, H-8), 7.48 (2H, d, *J* = 8.4 Hz, H-13), 7.98 (2H, d, *J* = 8.4 Hz, H-12), 8.12 (2H, d, *J* = 3.2 Hz, H-7), 9.13 (1H, br s, H-4); ^13^C-NMR (150 MHz, DMSO-*d*_6_) δ_C_ 21.1 (C-15), 39.2 (C-3), 47.9 (C-2), 108.0 (C-9a), 108.8 (C-8), 125.9 (C-8a), 128.3 (2C, C-12), 130.0 (2C, C-13), 133.1 (C-11), 133.2 (C-7), 133.3 (C-5a), 146.4 (C-14), 147.4 (C-4a), 166.3 (C-5), 172.9 (C-9); (−)-LRESIMS *m*/*z* (rel. int.) 405 (100) [M − H]^−^; (+)-LRESIMS *m*/*z* (rel. int.) 407 (100) [M + H]^+^; (+)-HRESIMS *m*/*z* 429.0167 [M + Na]^+^ (calcd for C_17_H_14_N_2_NaO_6_S_2_, 429.0185).

### 3.3. Deprotection of the N-Tosyl Regioisomer Mixture to Yield **13**

A 1:11 mixture of **12** and **11** (140 mg, 0.35 mmol) in a saturated solution of NaHCO_3_ (5 mL) and MeOH (50 mL) was heated to reflux for 2.5 h. The mixture was acidified with HCl (32% aqueous) to pH 6 then concentrated and subjected to flash chromatography (silica, 1:10 *v*/*v* MeOH/CH_2_Cl_2_ elution) to afford a 1:26 mixture of compounds **14** and **13** (59 mg, 68%). This material (59 mg) was pre-adsorbed to C_18_-bonded silica (1 g) overnight, then packed into a guard cartridge that was attached to a C_18_-bonded silica semi-preparative HPLC column. Application of the same reversed-phase HPLC purification method described above (Section 3.2) afforded **13** (15 mg, 11%, ^t^R = 23.0–24.0 min) as an orange powder.

Compound **13**: Orange amorphous solid; ^1^H-NMR (500 MHz, DMSO-*d*_6_) δ_H_ 3.31–3.33 (2H, m, H-2), 3.81–3.84 (2H, m, H-3), 6.57 (1H, d, *J* = 2.8 Hz, H-6), 7.13 (1H, d, *J* = 2.8 Hz, H-7), 9.05 (1H, brs, H-4), 12.76 (1H, brs, H-8); ^13^C-NMR (125 MHz, DMSO-*d*_6_) δ_C_ 39.4 (C-3), 48.2 (C-2), 107.5 (C-6), 120.7 (C-5a), 125.4 (C-7), 132.9 (C-8a), 148.2 (C-4a), 169.8 (C-9), 174.1 (C-5); (+)-LRESIMS *m*/*z* (rel. int.) 253 (100) [M + H]^+^; (−)-LRESIMS *m*/*z* (rel. int.) 251 (100) [M − H]^−^; (+)-HRESIMS *m*/*z* 275.0104 [M + Na]^+^ (calcd for C_10_H_8_N_2_NaO_4_S, 275.0097).

### 3.4. Synthesis of Regioisomers **13** and **14**

A solution of 1*H*-indole-4,7-dione (**10**, 504 mg, 3.4 mmol) in MeCN (160 mL) was treated with a solution of 2-aminoethanesulfinic acid (482 mg, 4.42 mmol) in H_2_O (50 mL) in one portion and the mixture stirred for 20 h under an atmosphere of O_2_. The MeCN and H_2_O were removed by rotary evaporation to afford an orange solid, which was purified by flash chromatography (silica, 1:5 *v*/*v* MeOH/CH_2_Cl_2_ elution) to afford a 3.3:1 mixture of regioisomers **14** and **13**, respectively (307 mg, 36%). A portion of this material (40 mg) was pre-adsorbed to C_18_-bonded silica (1 g) overnight, then packed into a guard cartridge that was attached to a C_18_-bonded silica semi-preparative HPLC column. Isocratic HPLC conditions of 95% H_2_O (0.1% TFA)/5% MeOH (0.1% TFA) were employed for the first 10 min, then a linear gradient to 50% H_2_O (0.1% TFA)/50% MeOH (0.1% TFA) was run over 40 min, followed by a linear gradient to MeOH (0.1% TFA) in 1 min, then isocratic conditions of MeOH (0.1% TFA) for a further 9 min, all at a flow rate of 9 mL/min. Sixty fractions (60 × 1 min) were collected by time from the start of the HPLC run. All UV active fractions were analyzed by ^1^H-NMR spectroscopy and MS, and identical fractions were combined. This yielded **14** (5.0 mg, 1%, ^t^R = 25.0–27.0 min) as a bright orange powder.

Compound **14**: Bright orange amorphous solid; ^1^H-NMR (500 MHz, DMSO-*d*_6_) δ_H_ 3.28–3.33 (2H, m, H-2), 3.79–3.82 (2H, m, H-3), 6.53 (1H, d, *J* = 2.5 Hz, H-8), 7.40 (1H, d, *J* = 2.5 Hz, H-7), 8.89 (1H, br s, H-4), 12.81 (1H, br s, H-6); ^13^C-NMR (125 MHz, DMSO-*d*_6_) *δ*_C_ 39.3 (C-3), 48.4 (C-2), 108.4 (2C, C-9a, C-8), 127.2 (C-8a), 128.2 (C-5a), 130.1 (C-7), 147.5 (C-4a), 167.9 (C-5), 174.7 (C-9); (+)-LRESIMS *m*/*z* (rel. int.) 253 (100) [M + H]^+^; (−)-LRESIMS *m*/*z* (rel. int.) 251 (100) [M − H]^−^; (+)-HRESIMS *m*/*z* 275.0087 [M + Na]^+^ (calcd for C_10_H_8_N_2_NaO_4_S, 275.0097).

### 3.5. Synthesis of Regioisomers **15** and **16**

A 3.3:1 regioisomeric mixture of **14** and **13** (90 mg, 0.36 mmol) from the synthesis described above (Section 3.4) in a solution of MeOH (10 mL) was treated with an aqueous KOH solution (6 mL, 12 M). The magnetically stirred reaction mixture was purged with O_2_, and maintained at 60 °C under a balloon of O_2_ for 4 h. The reaction mixture was cooled to 0 °C, carefully neutralized by the addition of an aqueous solution of HCl (1 M) and then the mixture was concentrated *in vacuo* to afford a residue that was subjected to flash chromatography through a small plug of silica (1:10 *v*/*v* MeOH/CH_2_Cl_2_ elution) and after concentration of the eluent *in vacuo*, the residue (40 mg) was pre-adsorbed to C_18_-bonded silica (1 g) overnight, then packed into a guard cartridge that was attached to a C_18_-bonded silica semi-preparative HPLC column. Application of same reversed-phase HPLC purification method described above (section 3.4) resulted in the purification of compounds **16** (15 mg, 17%, ^t^R = 28.0–30.0 min) and **15** (5 mg, 6%, ^t^R = 32.0–34.0 min) as yellow and orange powders, respectively.

Compound **15**: Orange amorphous solid; ^1^H-NMR (500 MHz, DMSO-*d*_6_) δ_H_ 6.49 (1H, d, *J* = 8.8 Hz, H-2), 6.66 (1H, d, *J* = 2.8 Hz, H-6), 7.07 (1H, d, *J* = 8.8 Hz, H-3), 7.27 (1H, d, *J* = 2.8 Hz, H-7), 11.07 (1H, br s, H-4), 12.99 (1H, br s, H-8); ^13^C-NMR (125 MHz, DMSO-*d*_6_) δ_C_ 108.0 (C-6), 111.7 (C-2), 113.4 (C-9a), 121.8 (C-5a), 126.8 (C-7), 130.2 (C-3), 131.3 (C-8a), 140.5 (C-4a), 172.2 (C-9), 174.1 (C-5); (+)-LRESIMS *m*/*z* (rel. int.) 251 (100) [M + H]^+^; (−)-LRESIMS *m*/*z* (rel. int.) 249 (100) [M − H]^−^; (+)-HRESIMS *m*/*z* 272.9939 [M + Na]^+^ (calcd for C_10_H_6_N_2_NaO_4_S, 272.9940).

Compound **16**: Yellow amorphous solid; ^1^H NMR (500 MHz, DMSO-*d*_6_) δ_H_ 6.42 (1H, d, *J* = 8.8 Hz, H-2), 6.60 (1H, d, *J* = 2.6 Hz, H-8), 7.04 (1H, d, *J* = 8.8 Hz, H-3), 7.43 (1H, d, *J* = 2.6 Hz, H-7), H-4 and H-6 not observed; ^13^C-NMR (125 MHz, DMSO-*d*_6_) δ_C_ 108.5 (C-8), 111.6 (C-2), 113.9 (C-9a), 126.9 (C-8a), 127.6 (C-5a), 129.8 (C-7), 130.1 (C-3), 140.2 (C-4a), 167.9 (C-5), 177.3 (C-9); (+)-LRESIMS *m*/*z* (rel. int.) 251 (100) [M + H]^+^; (−)-LRESIMS *m*/*z* (rel. int.) 249 (100) [M − H]^−^; (+)-HRESIMS *m*/*z* 272.9933 [M + Na]^+^ (calcd for C_10_H_6_N_2_NaO_4_S, 272.9940).

### 3.6. X-ray Crystallography Studies on Compound **11**

Intensity data were collected with an Oxford Diffraction SuperNova CCD diffractometer using Cu-Kα radiation, the temperature during data collection was maintained at 100.0(1) using an Oxford Cryosystems cooling device. The structure was solved by direct methods and difference Fourier Synthesis [13]. Hydrogen atoms bound to the carbon atom were placed at their idealized positions using appropriate HFIX instructions in SHELXL, and included in subsequent refinement cycles. Hydrogen atoms attached to nitrogen were located from difference Fourier maps and refined freely with isotropic displacement parameters. Thermal ellipsoid plots were generated using the program ORTEP-3 [14] integrated within the WINGX suite of programs [15]. Full details of the data collection and refinement and tables of atomic coordinates, bond lengths and angles, and torsion angles have been deposited with the Cambridge Crystallographic Data Centre (CCDC 1416796). Copies can be obtained free of charge on application at the following address: http://www.ccdc.cam.ac.uk.

Crystal data for compound **11**: C_17_H_14_N_2_O_6_S_2_, *M* = 406.42, *T* = 100.0(2) K, λ = 1.5418 Å, Triclinic, space group *P_21_/c*, *a =* 11.6802(7), *b =* 28.0975(14), *c =* 10.3047(6) Å, β = 91.539(5)° *V =* 3380.6(3) Å^3^, *Z* = 8, *Z*′ = 2, *D_c_* = 1.597 Mg·M^−3^, μ = 3.230 mm^−1^, *F(000)* = 1680, crystal size 0.49 mm × 0.38 mm × 0.31 mm. θ_max_ = 67.6°, 10,831 reflections measured, 5907 independent reflections (*R*_int_ = 0.051) the final *R* = 0.0559 [I > 2σ(I), 5134 data] and *w*R(F^2^) = 0.1577 (all data) GOOF = 1.027.

### 3.7. P. Falciparum Growth Inhibition Assay

*P. falciparum* growth inhibition assays were carried out using an isotopic microtest, as previously described [16]. Briefly, *in vitro* cultured *P. falciparum* infected erythrocytes (1.0% parasitemia and 1.0% hematocrit) were seeded into triplicate wells of 96 well tissue culture plates containing vehicle control (DMSO), positive control [chloroquine (Sigma-Aldrich, St. Louis, MO, USA), catalogue #C6628, >98%] or test compounds and incubated under standard *P. falciparum* culture conditions with 0.5 μCi [^3^H]-hypoxanthine. The final concentration of DMSO vehicle was <0.5% in all assay wells (non-toxic). After 48 h cells were harvested onto 1450 MicroBeta filter mats (PerkinElmer, Waltham, Massachusetts, USA) and [^3^H] incorporation determined using a 1450 MicroBeta liquid scintillation counter. Percentage inhibition of growth compared to matched DMSO controls was determined and IC_50_ values were calculated using linear interpolation of inhibition curves [17]. The mean IC_50_ or % inhibition (±SD) was calculated for three independent experiments, each carried out in triplicate.

## 4. Conclusions

In summary, six analogues associated with the tricyclic core of thiaplakortones were synthesized from readily accessible and known 1*H*-indole-4,7-dione derivatives, and isolated in low to moderate yields. Regiochemistry was moderated by substitution of the indole nitrogen. All compounds were tested for *in vitro* antiplasmodial activity towards two *P. falciparum* parasite lines (3D7 and Dd2). Compound **16** showed the best antiparasitic activity with IC_50_ values of 252 and 127 nM towards 3D7 and Dd2 lines, respectively. The moderate toxicity (IC_50_ 4600 nM) of compound **16** towards NFF cells equates to a selectivity index of 18.2–36.2. These studies have identified that while the ethylamine side-chain present in the marine natural products, thiaplakortones A and B, translates to more potent and selective antiplasmodial compounds, this functionality is by no means essential for activity. Furthermore, the truncated thiaplakortone molecules (**11**–**16**) synthesized during this work has allowed delineation of a minimum antiplasmodial pharmacophore for the thiaplakortone chemotype.

## Supplementary Files

Supplementary File 1
