# Supplementary material for: Synthesis and Antiplasmodial Evaluation of Analogues Based on the Tricyclic Core of Thiaplakortones A–D
Source: Mar Drugs. 2015 Sep 15;13(9):5784–95. doi: 10.3390/md13095784 (PMC4584354; doi:10.3390/md13095784)
Supplement: Supplementary File 1 [file marinedrugs-13-05784-s001.docx]

Supplementary Materials

General Experimental Procedures

Unless otherwise specified, ^1^H and ^13^C NMR spectra were recorded at 30 °C in DMSO-*d*6 on Varian INOVA 500 or 600 MHz NMR spectrometers. The latter spectrometer was equipped with a triple resonance cold probe. The ^1^H and ^13^C NMR chemical shifts were referenced to the solvent peak for DMSO-*d*6 at δ_H_ 2.50 and δ_C_ 39.5.


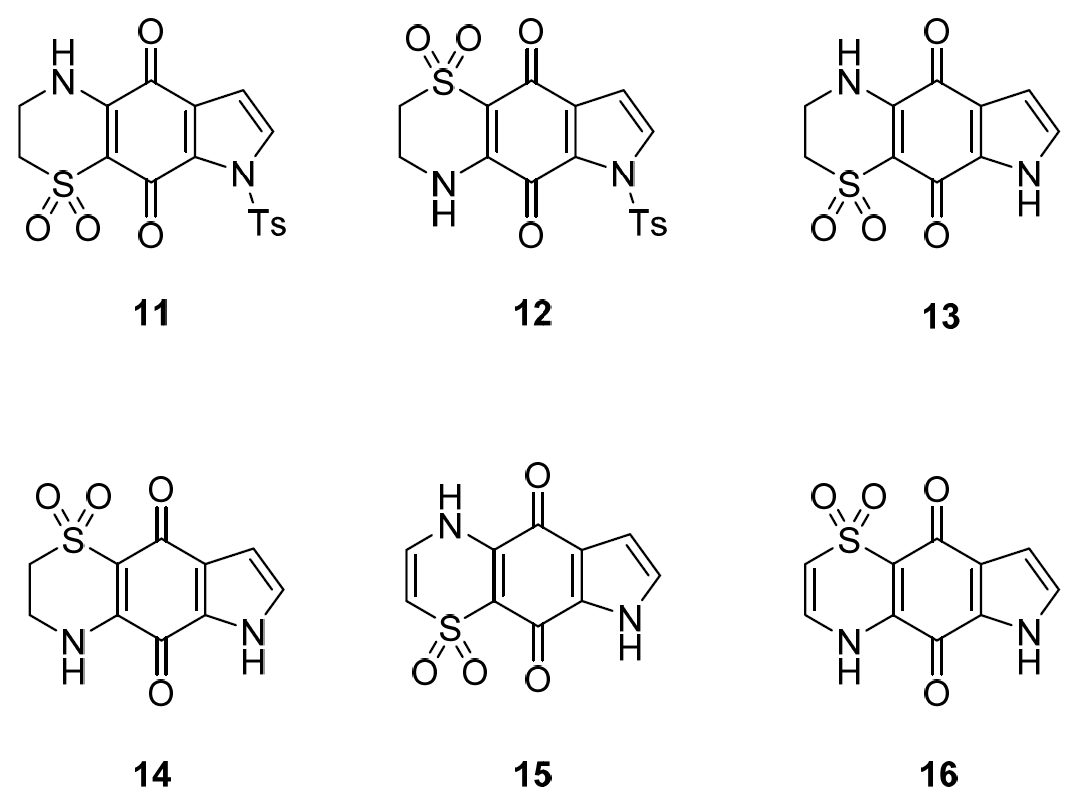


**Chart 1.** Structure of compounds **11**–**16**.


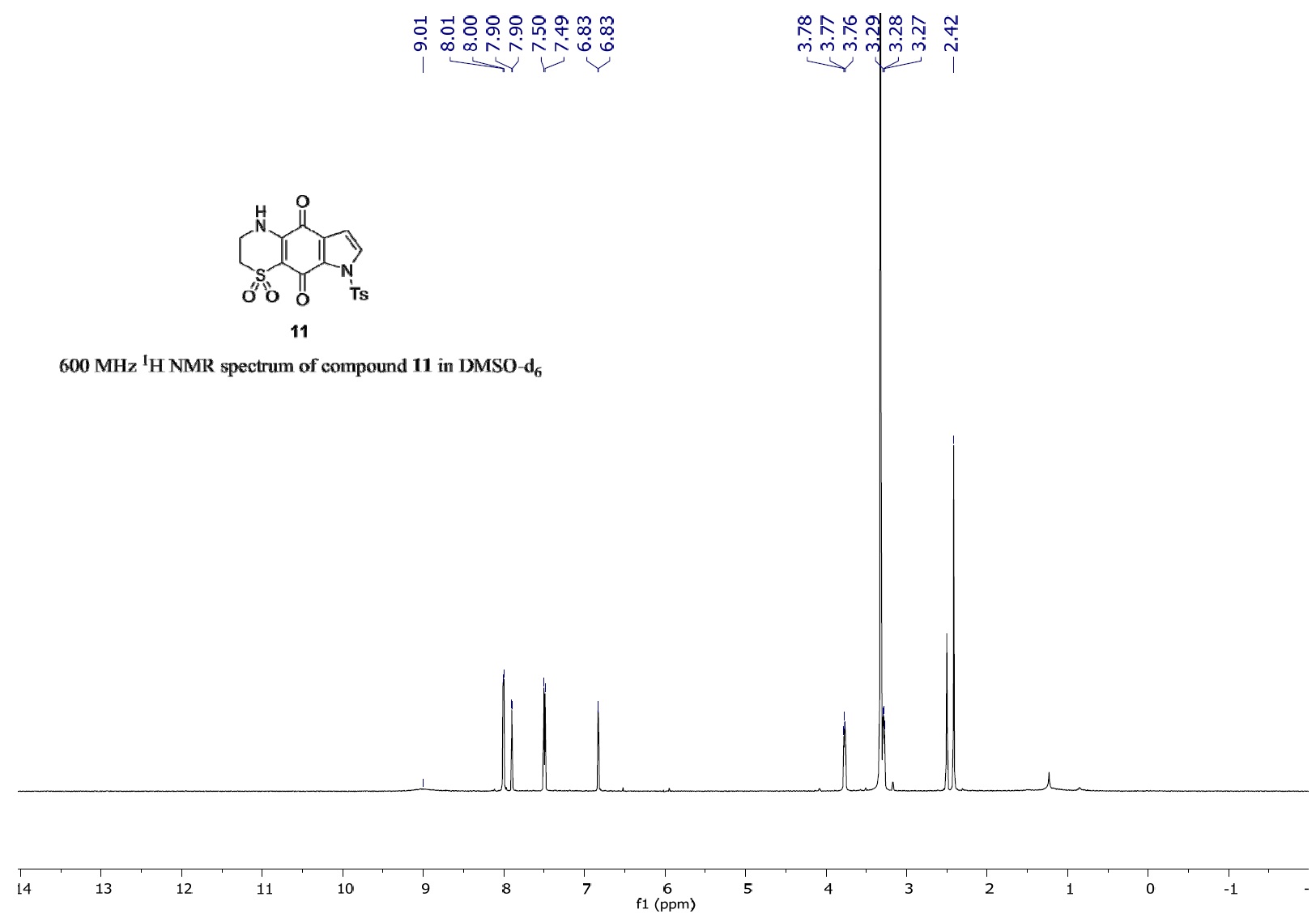


**Figure S1.** 600 MHz ^1^H NMR spectrum of compound **11** in DMSO-*d*6.


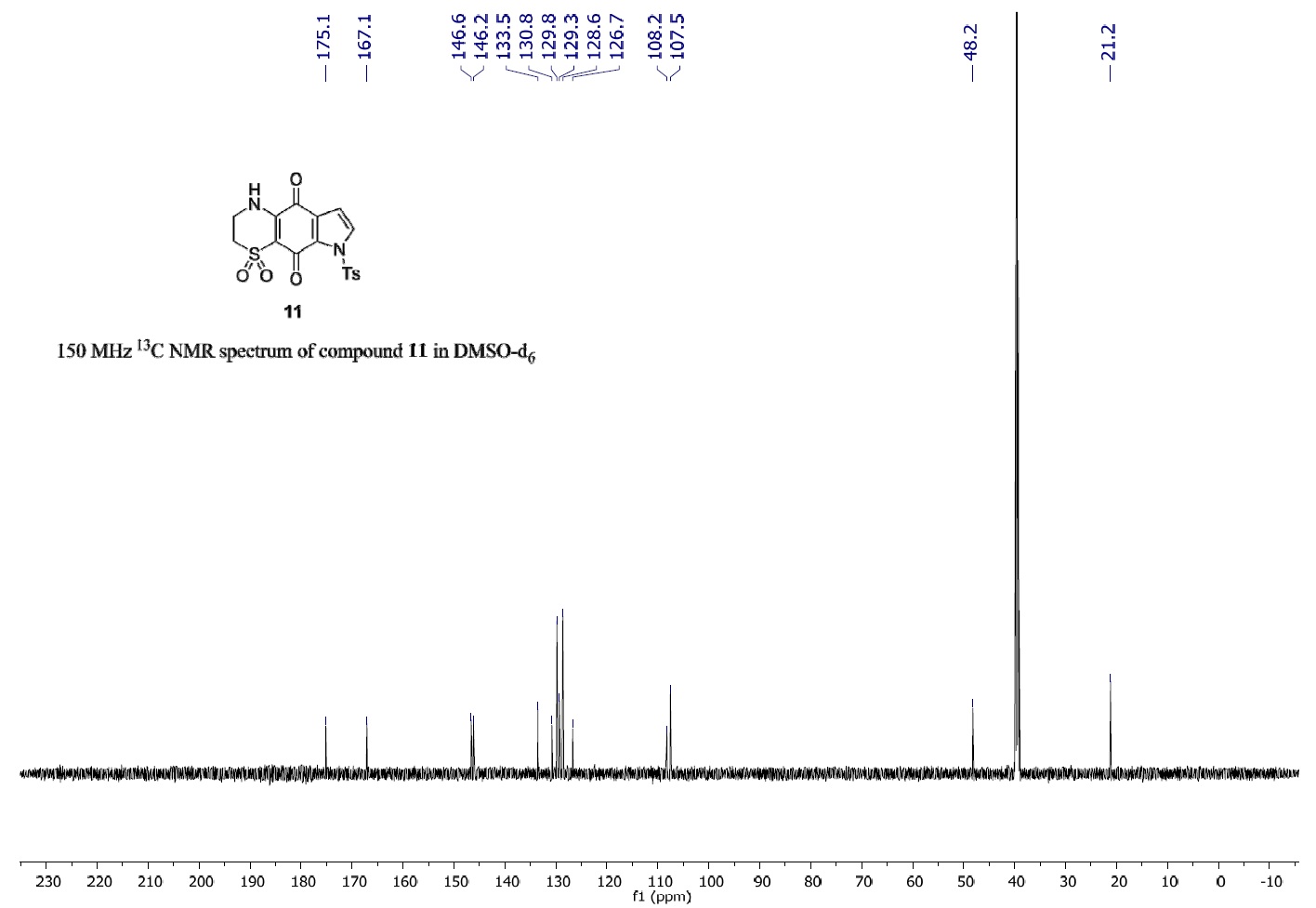


**Figure S2.** 150 MHz ^13^C NMR spectrum of compound **11** in DMSO-*d*6.


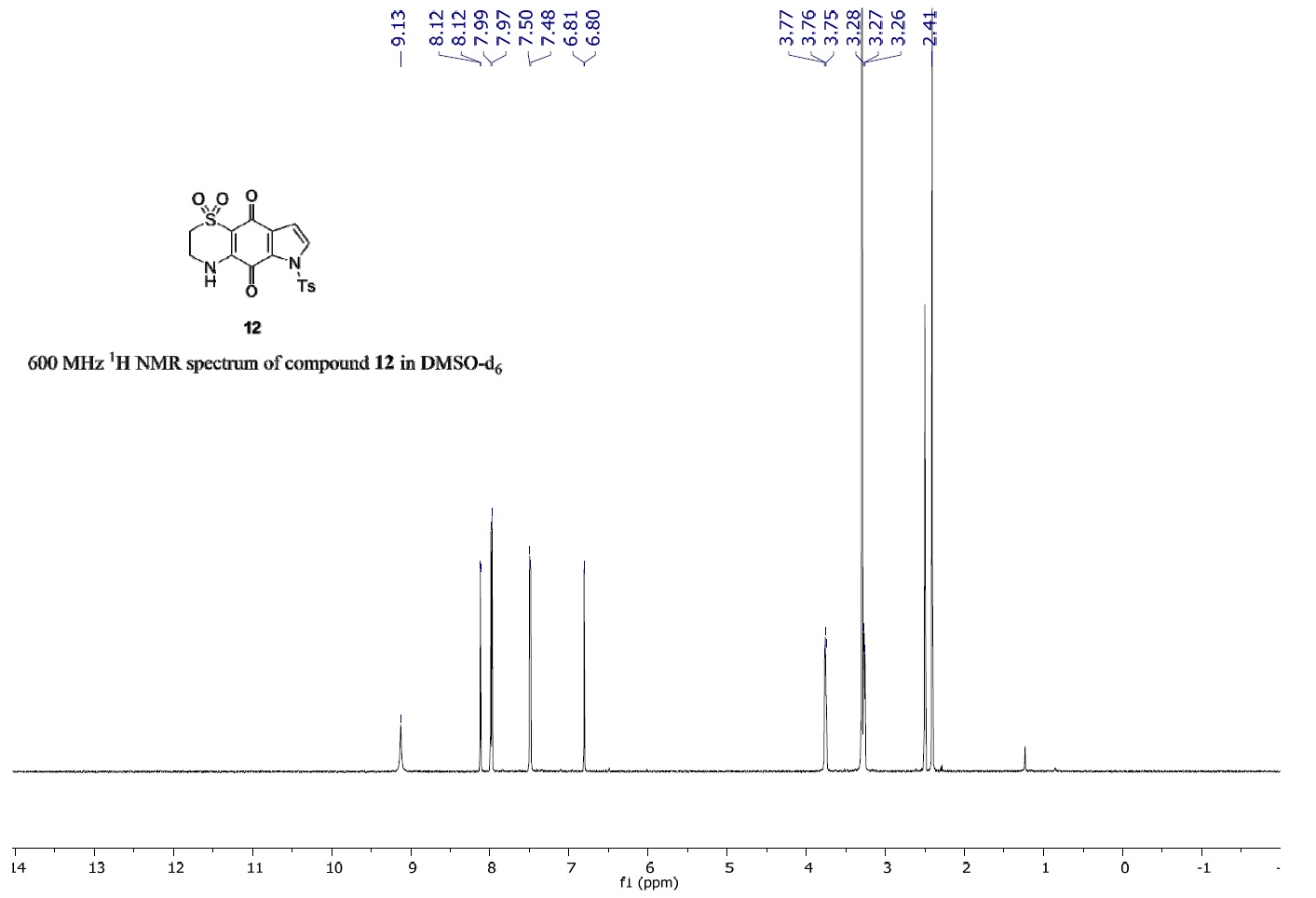


**Figure S3.** 600 MHz ^1^H NMR spectrum of compound **12** in DMSO-*d*6.


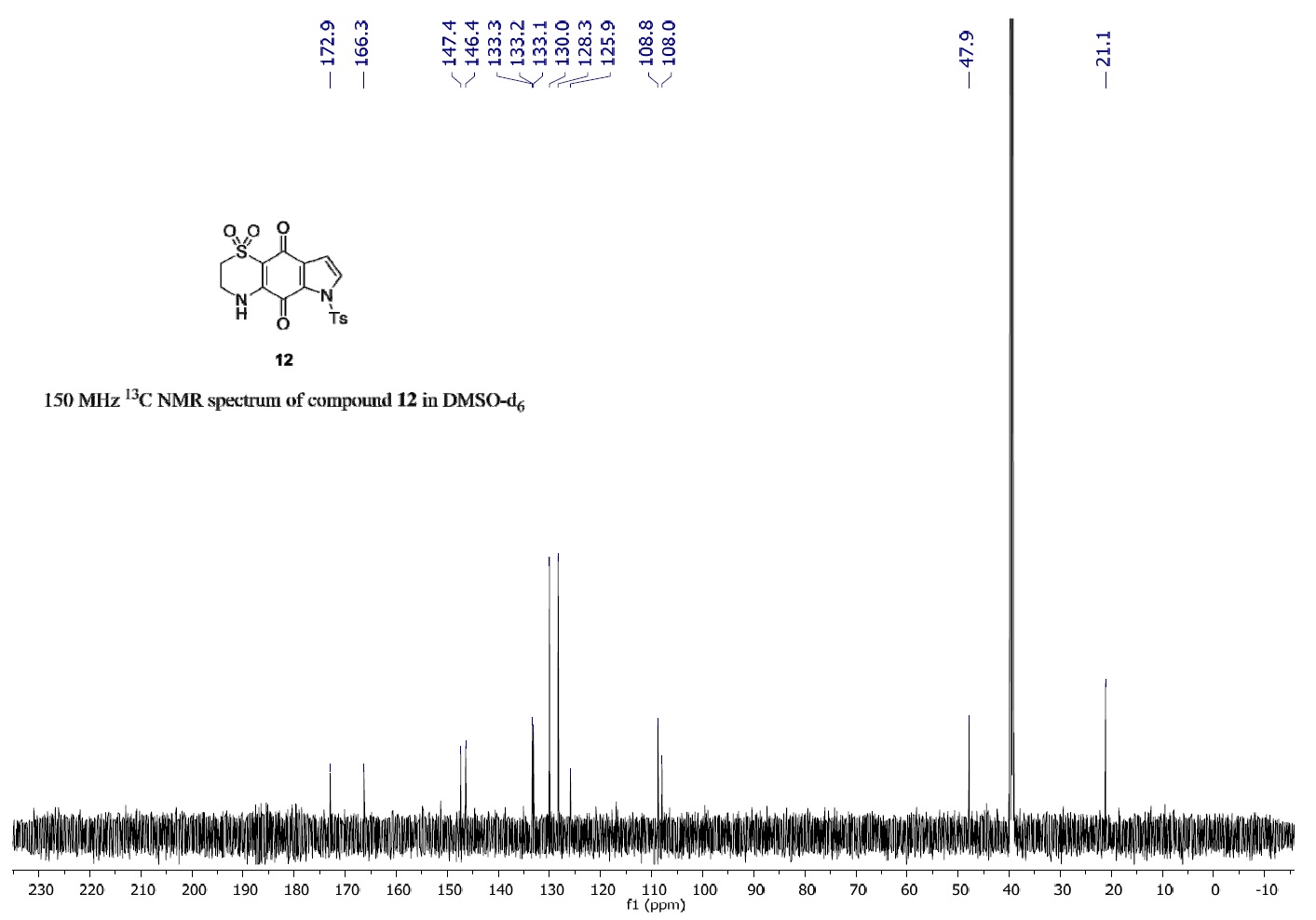


**Figure S4.** 150 MHz ^13^C NMR spectrum of compound **12** in DMSO-*d*6.


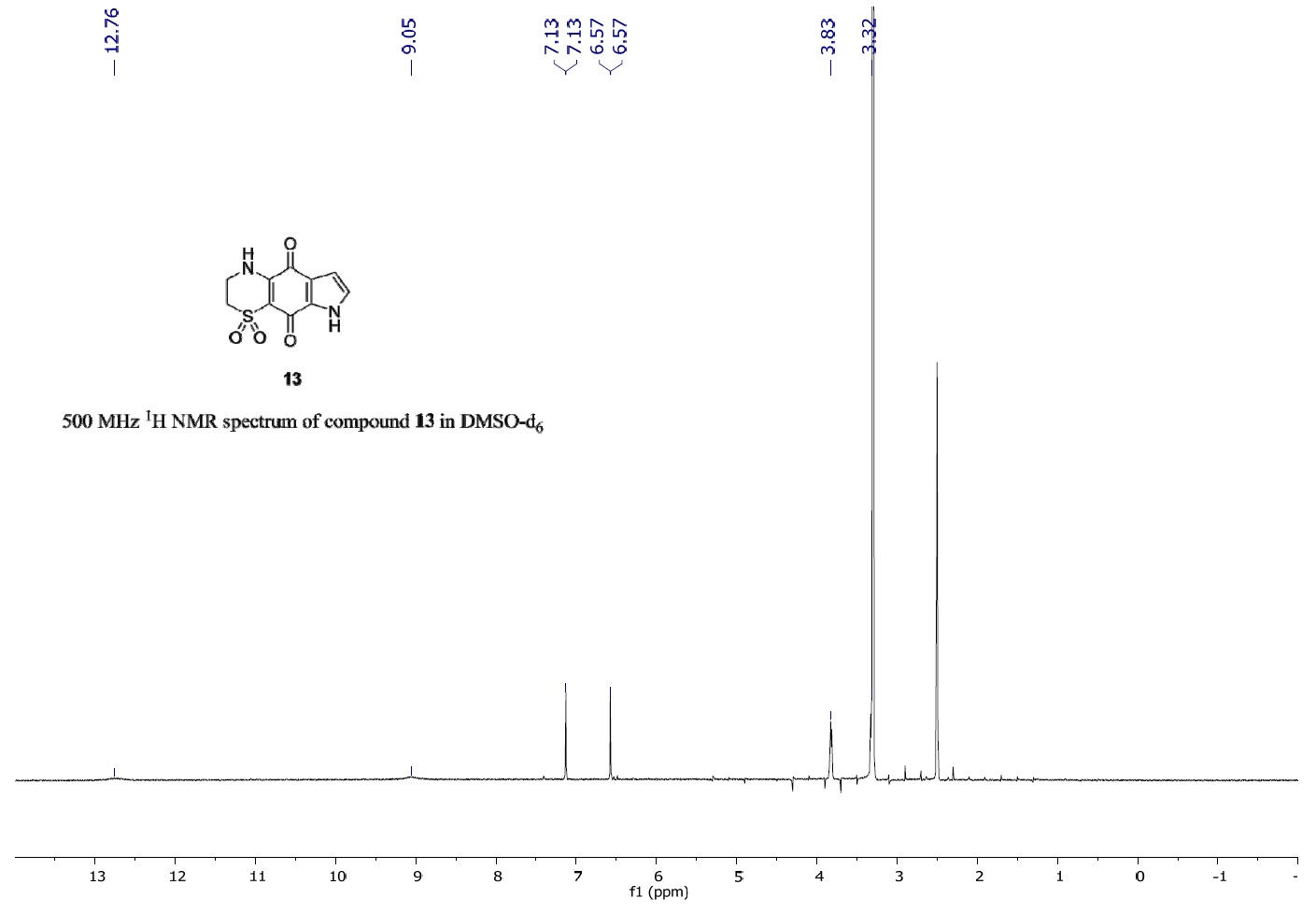


**Figure S5.** 500 MHz ^1^H NMR spectrum of compound **13** in DMSO-*d*6.


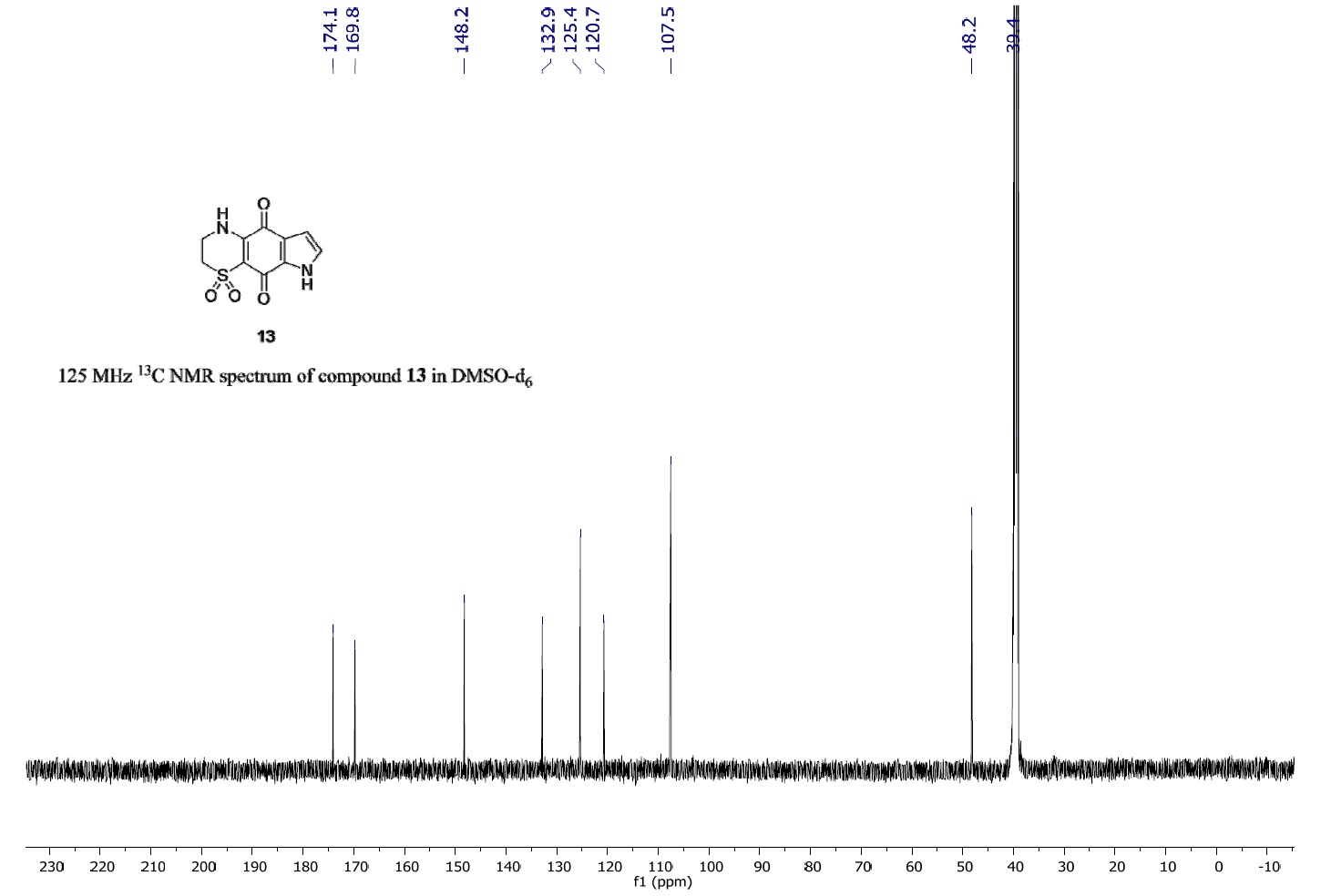


**Figure S6.** 125 MHz ^13^C NMR spectrum of compound **13** in DMSO-*d*6.


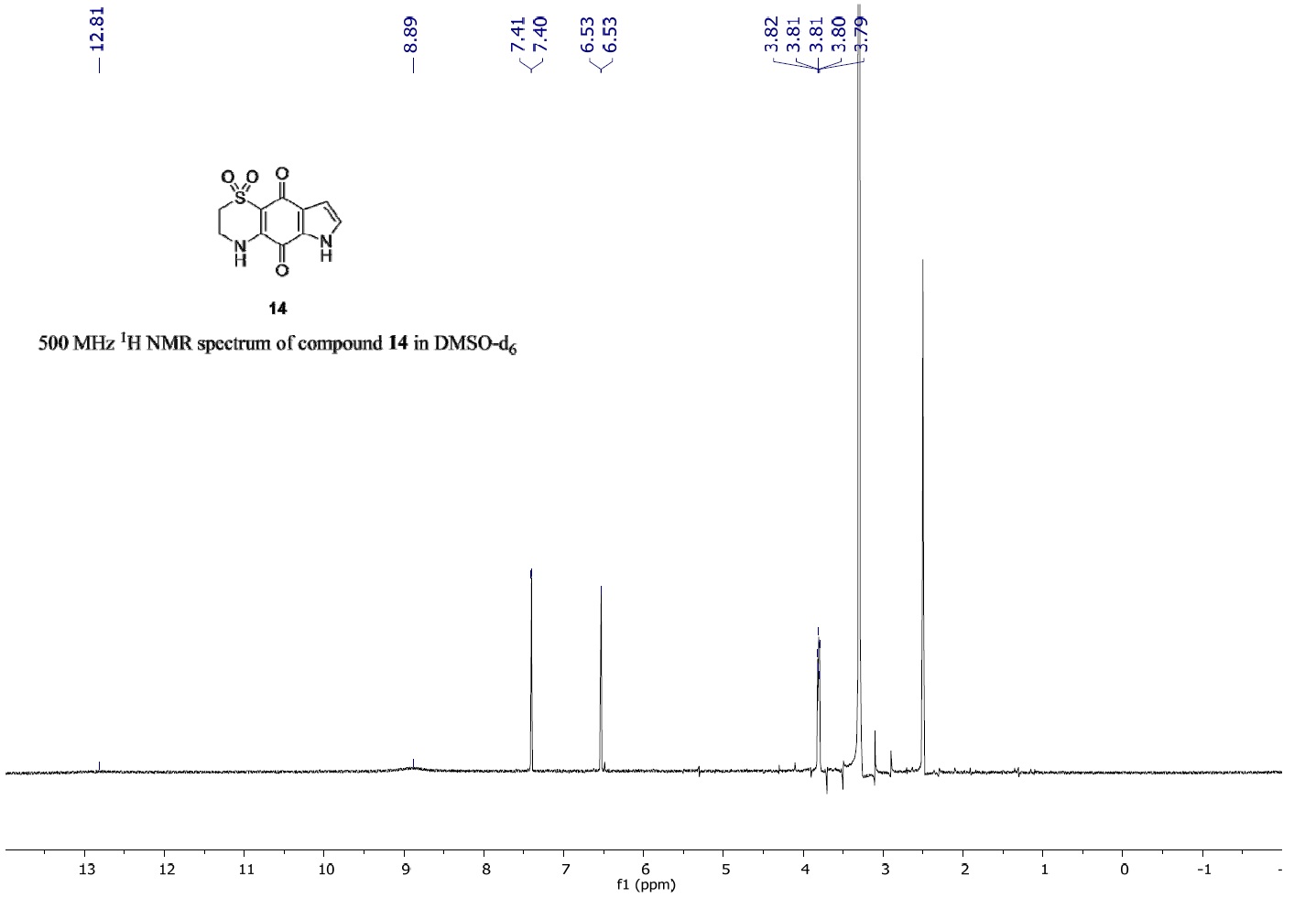


**Figure S7.** 500 MHz ^1^H NMR spectrum of compound **14** in DMSO-*d*6.


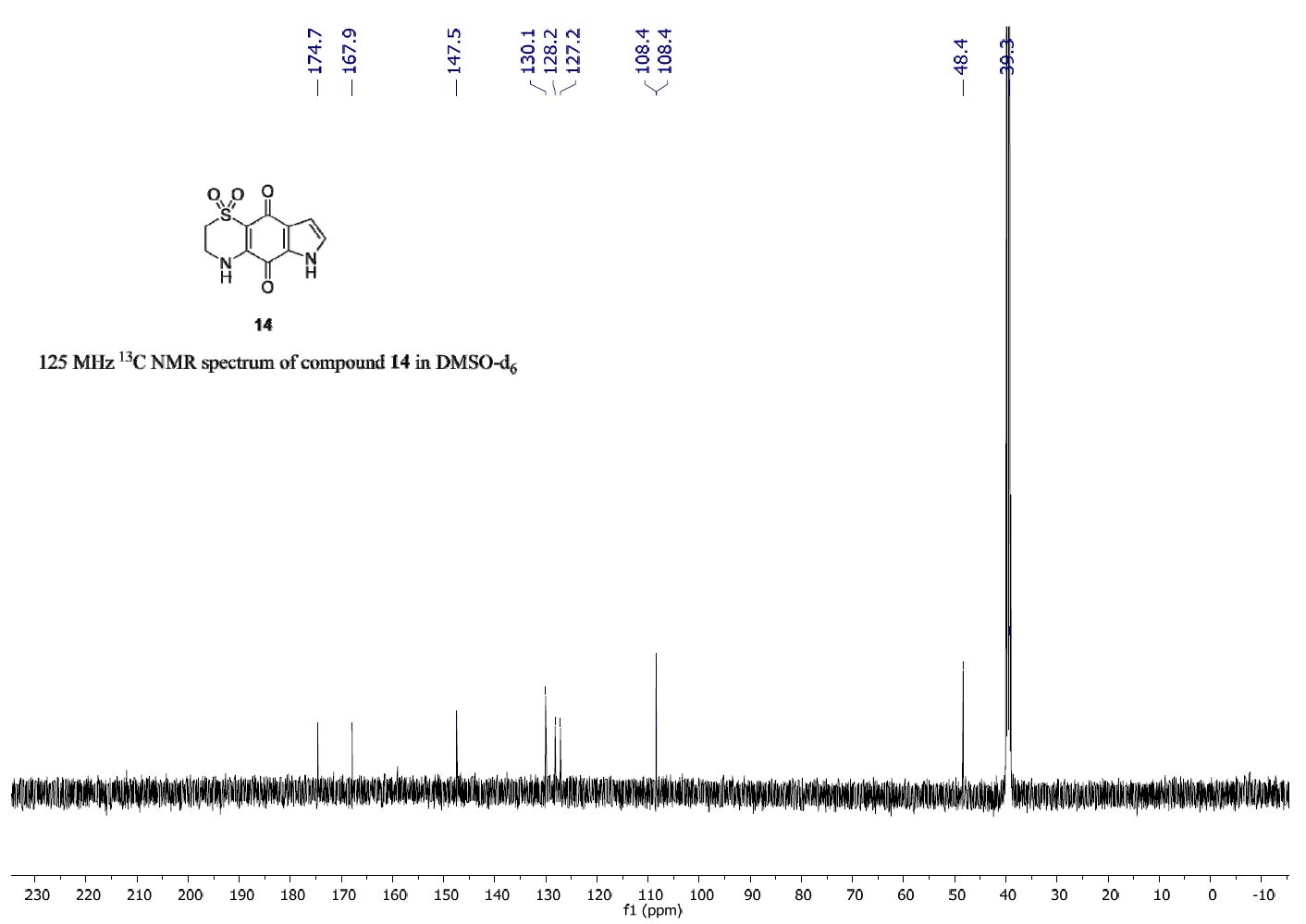


**Figure S8.** 125 MHz ^13^C NMR spectrum of compound **14** in DMSO-*d*6.


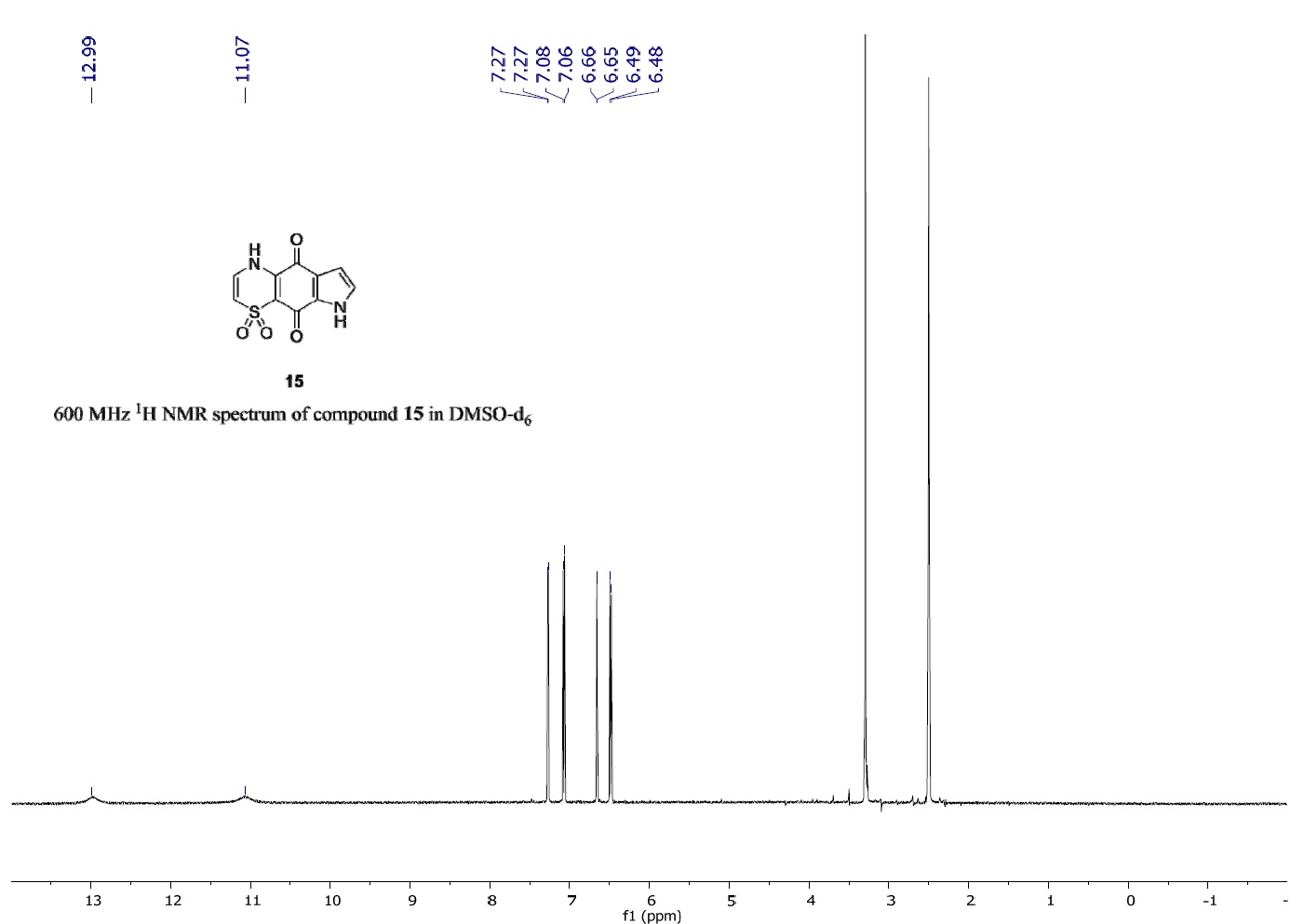


**Figure S9.** 600 MHz ^1^H NMR spectrum of compound **15** in DMSO-*d*6.


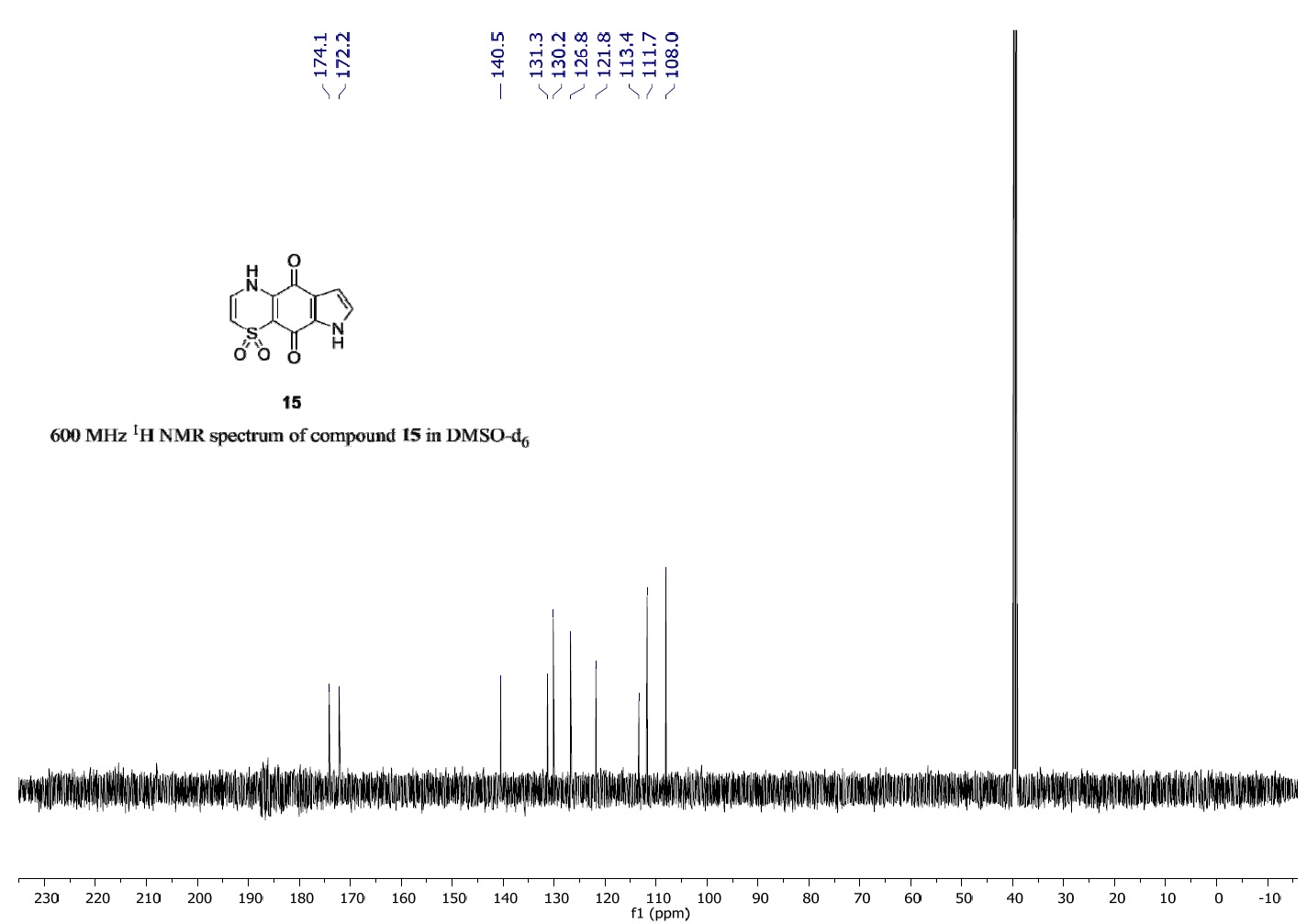


**Figure S10.** 600 MHz ^1^H NMR spectrum of compound **15** in DMSO-*d*6.


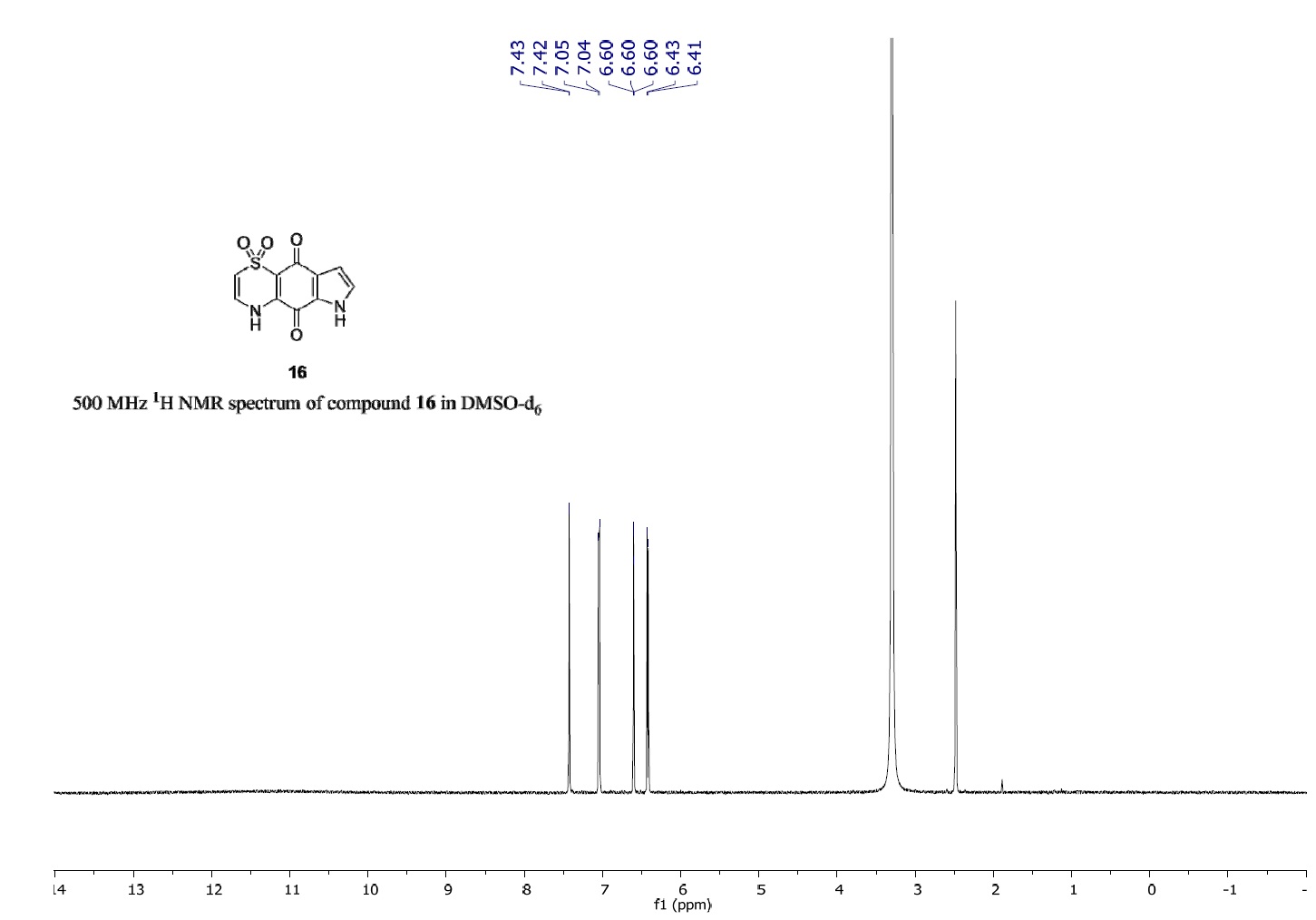


**Figure S11.** 500 MHz ^1^H NMR spectrum of compound **16** in DMSO-*d*6.


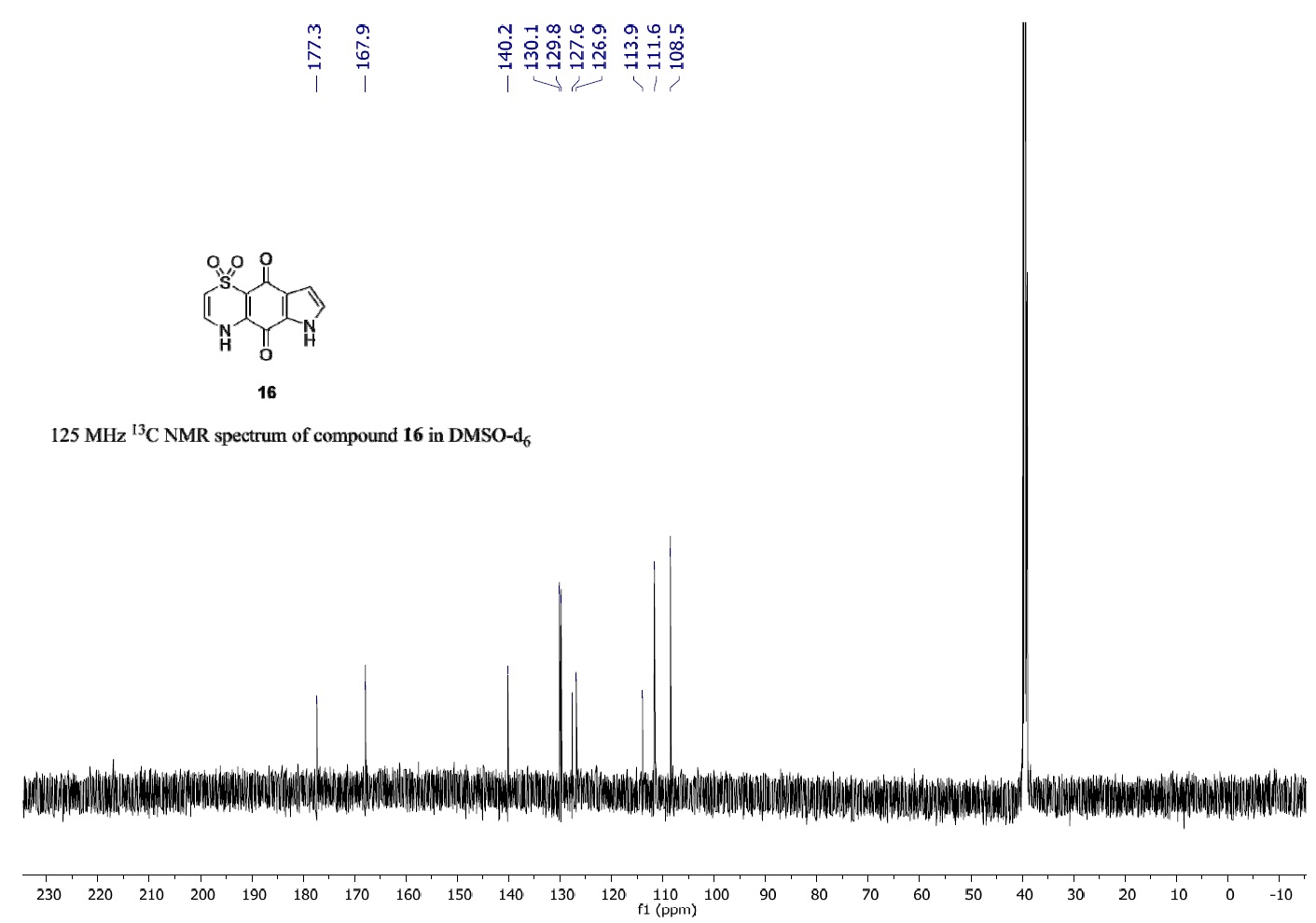


**Figure S12.** 125 MHz ^13^C NMR spectrum of compound **16** in DMSO-*d*6.

X-ray Crystallographic Data for Compound 11


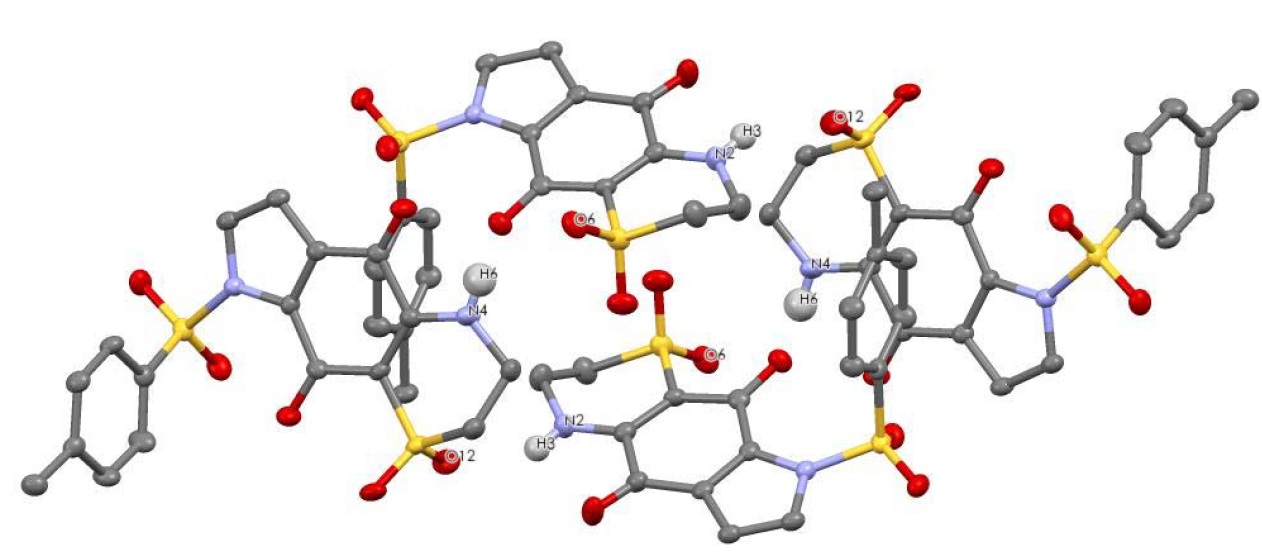


**Figure S13.** Tetrameric cluster of compound held together by N–H···O hydrogen bonds.

The molecules of 11 are held together by N–H···O intermolecular hydrogen bonds forming a
H-bonded tetramer (Figure S13), the H-bond parameters are summarized in Table S1.

**Table S1.** Hydrogen bond parameters for Compound 11 [Å and °].

| **D–H···A** | **d(D–H)** | **d(H···A)** | **d(D···A)** | **<(DHA)** |
| --- | --- | --- | --- | --- |
| N(2)–H(3) ···O(12)#1 | 0.81(4) | 2.28(4) | 3.019(4) | 151(4) |
| N(4)–H(6) ···O(6)#2 | 0.82(5) | 2.35(5) | 2.965(4) | 132(4) |

Symmetry transformations used to generate equivalent atoms: #1 −x+2, −y, −z; #2 x+1, y, z; #3 x, −y+1/2, z−1/2; # 4 x+1, −y+1/2, z+1/2; #5 −x+2, −y, −z+1.

© 2015 by the authors; licensee MDPI, Basel, Switzerland. This article is an open access article distributed under the terms and conditions of the Creative Commons Attribution license (http://creativecommons.org/licenses/by/4.0/).
